# Into the Seed: Auxin Controls Seed Development and Grain Yield

**DOI:** 10.3390/ijms21051662

**Published:** 2020-02-28

**Authors:** Jinshan Cao, Guoji Li, Dejie Qu, Xia Li, Youning Wang

**Affiliations:** State Key Laboratory of Agricultural Microbiology, College of Plant Science and Technology, Huazhong Agricultural University, Wuhan 430070, China; jinshancao@webmail.hzau.edu.cn (J.C.); lgj2389868525@163.com (G.L.); dejiequ@163.com (D.Q.); xli@mail.hzau.edu.cn (X.L.)

**Keywords:** auxin, auxin metabolism, auxin signaling, seed development, seed weight, seed yield

## Abstract

Seed development, which involves mainly the embryo, endosperm and integuments, is regulated by different signaling pathways, leading to various changes in seed size or seed weight. Therefore, uncovering the genetic and molecular mechanisms of seed development has great potential for improving crop yields. The phytohormone auxin is a key regulator required for modulating different cellular processes involved in seed development. Here, we provide a comprehensive review of the role of auxin biosynthesis, transport, signaling, conjugation, and catabolism during seed development. More importantly, we not only summarize the research progress on the genetic and molecular regulation of seed development mediated by auxin but also discuss the potential of manipulating auxin metabolism and its signaling pathway for improving crop seed weight.

## 1. Introduction

With the exponential increase in the global population, food supplies have become a serious issue that cannot be ignored. The critical question is how to sustain food production for the planet but without any additional increases in the use of available arable land. Importantly, the optimization and utilization of the main factors influencing crop yields have great potential for increasing yields in the limited areas with soil. Among these factors, seed weight is one of the most important three factors determining grain production of cereal crops. Accordingly, bulk seed weight is used as an indicator of average seed size [1], and this morphophysiological trait contributes to seed development in domesticated plant species [2,3].

Seed development is coordinated by the growth of the embryo, endosperm and the maternal ovule in both monocots and dicots, in which the maternal ovule develops into the integuments and ultimately leads to the generation of the seed coat [2,4,5]. In many dicots, both integument (seed coat) and embryo development play determinative roles in seed size. Except for supplying nutrients for embryo development, most of the endosperm volume is replaced by the embryo [6]. In contrast, in monocots and some dicots, the endosperm is retained and contributes to the volume of the mature seed [2]. Interestingly, the growth of the seed is not primarily related to the subsequent growth of the embryo but rather to the initial growth of the endosperm [2]. In addition, in some dicot species, such as those of Arabidopsis and Brassica, the silique wall has been confirmed to be the main source of nutrition in developing seeds, providing photosynthates for seed growth [7,8,9,10]. Various cellular processes also influence seed development. Endosperm formation occurs initially via nuclear divisions and subsequent cellularization, and the endosperm grows much more rapidly than the embryo. Due to the retaining of a large endosperm, the process of endosperm cellularization is also involved in seed size control [11].

During the past century, seed or grain size has been considered an important indicator for breeders; however, only within the past decade have the molecular regulators mediating seed size formation been started to be identified in plants, particularly in model plant species, such as Arabidopsis [5,11,12,13,14,15,16,17,18,19]. Several signaling pathways that determine seed size by affecting the growth of maternal tissue and/or endosperm have been defined, including the IKU (HAIKU) pathway, the ubiquitin-proteasome pathway, G-protein signaling and multiple phytohormone signaling pathways [5,13,17,18,20,21,22,23,24,25,26,27,28,29,30,31]. Thus, seed development is controlled by spatially and temporally integrated molecular regulatory networks coupled with the spatial-temporal distribution of multiple types of phytohormones. 

**Figure 1:**
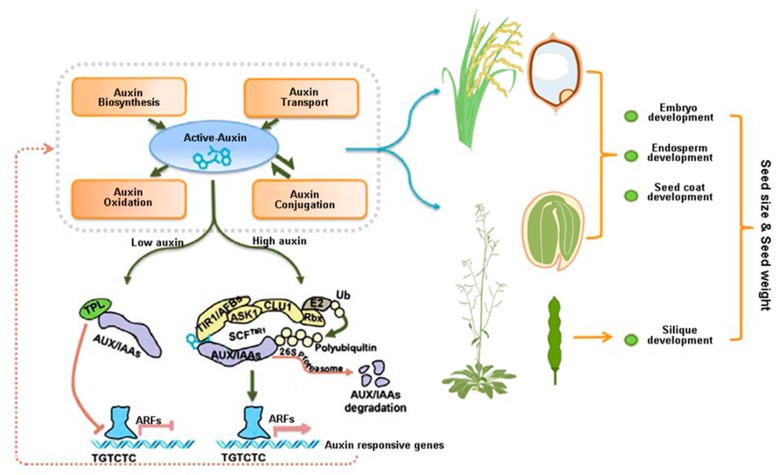
The developmental processes that are controlled by auxin and its potential impacts on seed size and weight. Active auxin, influenced by auxin biosynthesis, oxidation, conjugation, and transport, initiates auxin signaling pathway, in turn, which also have a potential feedback loop for maintaining the fine cellular auxin concentration. In both monocotyledon and dicotyledon plants, auxin has been found to be involved in regulating the development of embryo, endosperm and seed coat, which have the potential role in determining the seed size and seed weight of plants. Recent studies have confirmed that silique development also has the impact on seed development.

Among these hormones, auxin is a key component in seed development and seed weight. The role of auxin in seed development was noticed approximately 80 years ago [32], and compelling evidence has demonstrated that auxin regulation of seed development is concentration dependent. The spatial-temporal distribution of active auxin is dynamically modulated by auxin biosynthesis, auxin polar transport and signal transduction, auxin conjugation, and auxin catabolism, all of which maintain auxin at optimal levels for seed development. In this review, we provide an extensive overview of the role of auxin during seed development (Table 1, Figure 1) and discuss the potential significance in increasing crop yields.

## 2. Auxin Levels Influence Seed Development

It is well known that auxin regulates various aspects of seed development, including the development of the embryo, endosperm and seed coat, after fertilization. In plants, auxin accumulation and distribution are varied during seed development. It has been shown that, in maize, the concentration of free indole-3-acetic acid (IAA) quickly increases between 8 and 28 days after pollination (DAP), with a decline at 20 DAP [33,34,35]. In Arabidopsis, auxin accumulates in immature seeds at the heart, torpedo, and cotyledon stages, specifically at the ends of hypophysis and cotyledon primordia during somatic embryo development [36]. The endogenous IAA levels in the spikelets also increased after pollination and during subsequent fruit development, and this increase is positively correlated with IAA synthesis in the ovary of rice [37]. There is evidence to show that OsGE/CYP78B5 may regulate embryo size by maintaining normal levels of IAA in rice [38]. In line with these results, auxin has been found to be involved in regulating endosperm proliferation in maize [33,39]. Recent studies have confirmed that auxin production also influences endosperm proliferation rates and cellularization during seed development in Arabidopsis [40,41] Interestingly, auxin synthesized in the developing endosperm can be exported into the integuments and is necessary for seed coat development [40,42]. These observations support that auxin is required for regulating embryo, endosperm, and seed coat development.

The main source of auxin originates from its biosynthesis. To date, five IAA biosynthetic pathways have been proposed, including four inter-connected Trp-dependent IAA biosynthetic pathways and one Trp-independent pathway [35,43]. Among these pathways, the indole-3-pyruvic acid (IPA) pathway has been found to be the main IAA biosynthesis pathway in Arabidopsis, in which both TAA (tryptophan amino transferases) and YUCCA (YUC) function as main components that modulate this pathway [44,45,46]. Previous studies have shown that *TAA*- and *YUC*-related genes participate in regulating seed development. In Arabidopsis, *YUC1*, *YUC4*, *YUC10*, and *YUC11* redundantly regulate embryonic development by modulating auxin biosynthesis at the globular stage [47]. In pea, *tar2-1*, a loss-of-function mutant of *PsTAR2 (TRYPTOPHAN AMINOTRANSFERASE-RELATED 2)*, presents reduced embryo fresh weight (FW), leading to the formation of a small seed with a reduced starch content and a wrinkled phenotype [43,48]. Interestingly, some evidence shows that maternally produced auxin in the integuments is required for early embryo development of Arabidopsis [49]. Auxin biosynthesis is also required for endosperm development. It has been reported that the MADS-box transcription factor AGL62 (AGAMOUS-LIKE 62) contribute to endosperm initiation through repressing auxin biosynthesis genes expression [40]. A loss of mutation in the *Defective Endosperm 18/ZmYuc1* gene (*DE18/ZmYuc1*) is associated with IAA deficiency, leading to defective proliferation of the endosperm and a small-seed phenotype [33,50]. Additionally, it is interesting to note that auxin also regulates silique development. Overexpression of *BnaA9.CYP78A9*, which encodes a P450 monooxygenase, induces a significant increase in auxin in developing siliques, stimulating the elongation of siliques in *Brassica napus* [51].

Although auxin has an important role in regulating seed development, the regulatory mechanisms that underlie auxin-modulated auxin synthesis and accumulation have received little research attention. Until now, only a few findings have given clues about the regulatory mechanism governing auxin. It has been found that, to modulate seed development, the key genes involved in auxin biosynthesis might be directly regulated by transcription factors. For example, *YUC4* has been confirmed to be a direct target of LEC2 during somatic embryogenesis [52,53]. The fact that one of MADS-box transcription factors, MADS29, a key regulator in endosperm development, is also induced by auxin in rice suggests alterations in auxin during endosperm development [54]. These data suggest that auxin biosynthesis might undergo transcriptional regulation during seed development.

Auxin also has the capability of regulating apomictic seed formation (apomixis), which has high economic potential for maintaining or utilizing heterosis by permitting the formation of seeds without fertilization [55]. It has long been noted that exogenous applications of auxin can induce parthenocarpic fruit development by stimulating the growth of ovaries in plant species such as tomato, petunia, salpiglossis, and pepper [32]. In other species, exogenous applications of synthesized auxin (2,4-D) can also promote parthenocarpic development of rice ovaries [37,56] and stimulate autonomous endosperm proliferation and seed coat development in maize and Arabidopsis [34,40,42]. The phenotypic effects of the *TAR2* mutation could be partially rescued by 2,4-D applications to mature pea leaves [48]. These results indicate the potential role of auxin in crop breeding in which heterosis is maintained via engineered apomixis [55,57,58]

## 3. Auxin Transport-Mediated Seed Development

Auxin transport is responsible for the auxin redistribution and gradient in different plant tissues in response to developmental signals or environmental stimuli. PIN-FORMED (PIN) efflux transporters and AUXIN/LIKE AUXIN (AUX/LAX family) auxin influx carriers (AUX/LAX1 family) are responsible for auxin distribution in plant cells [59]. Extensive evidence has shown that auxin efflux and influx mediate the active transport of auxin during seed development.

Auxin-dependent cell specification involved in embryo development requires the balance of auxin transport modulated by both influx and efflux mechanisms [60]. It has been reported that polar auxin transport is correlated with embryonic differentiation and definition [39]. Moreover, it has been assumed that PIN-mediated auxin efflux is responsible for seed development, which is also supported by the results of expression patterns and morphological analyses. In Arabidopsis, there are 8 *PIN-FORMED (PIN)* genes that encode auxin efflux transporters and control polar auxin transport in plants [9,35,61], among which *PIN1*, *PIN3*, *PIN4*, and *PIN7* are expressed in the embryo [62]. Phenotypic analysis showed that pin1 mutants display defects at the basal embryo pole, while the *pin7* mutant appears to have defects in the stereotypical patterning of early embryogenesis [62]. Moreover, the quadruple mutant *pin1 pin3 pin4 pin7* displays severe defects in proembryo establishment, indicating functional redundancy among different PIN proteins [62]. Additional studies have confirmed that an apical-basal auxin gradient regulated by PIN1 and PIN7 appears to function in specifying the apical embryo structures and subsequently reorganizing the auxin gradient for specification of the basal root pole [62,63]. In line with this result, previous studies in wheat support the notion that heterogeneous auxin distribution has a conserved role in modulating embryonic pattern formation [64,65]. In addition, the expression patterns of homologous *ZmPIN* genes appear to be different during kernel development. The expression of three *ZmPIN1* genes is induced after double fertilization, and their associated proteins also colocalize in developing embryos [39]. In accordance with these results, the expression of *ZmPIN5c* is upregulated from 3 to 12 DAP during kernel development [66]. Although there is the possibility for functional redundancy among PIN proteins, these findings also provide strong evidence for the conserved role of auxin polar transport mediated by PIN proteins during seed development. Accordingly, treatment of kernels with the auxin transport inhibitor N-1-naphthylphthalamic acid (NPA) abolishes the auxin gradient inside the embryo and the relatively high accumulation of auxin in the embryo root during the morphogenetic phase, leading to abnormal embryonic root development [39]. At the early stages of endosperm development, auxin appears to highly accumulate at the endosperm margin but is relatively low in the center of the endosperm [35], which is disrupted by the addition of NPA, resulting in a multilayered aleurone [39]. Moreover, *Medicago truncatula DASH*, encoding an endosperm-specific DOF transcription factor, is also identified to positively regulate endosperm development by affecting auxin export [67]. 

Studying the role of PIN proteins (auxin efflux transporters) during seed development has been gaining increased amounts of attention. By contrast, the role of auxin influx carriers in seed development remains elusive. It has been well documented that AUX1 (AUXIN RESISTANT 1) and its homologues LAX1, LAX2, and LAX3 (LIKE AUXIN RESISTANT) participate in auxin cellular influx [68,69,70]. Members of the AUX1/LAX family have been found to be associated with the establishment of cell patterns in the apex of embryonic roots [71]. A convincing example comes from phenotypic analysis of a double mutant, aux1 lax, which has a larger radicle root cap than does the wild type [71]. In contrast, the embryonic phenotype of *aux1 lax1 lax2 lax3* quadruple mutants exhibits an extreme disorganization of the radicle apex [71]. Until recently, it has been demonstrated that AUX1, LAX1, and LAX2 are involved in the formation of the shoot and root poles in both the microspore-derived embryos of *Brassica napus* and the zygotic embryos of Arabidopsis thaliana [60]. These results provide direct evidence that the auxin importers AUX1/LAX are also regulators of embryonic root formation. 

## 4. The Auxin Signaling Pathway Is Involved in Seed Development 

The fluctuation of auxin accumulation can trigger or deactivate the auxin signaling pathway, which can mediate cellular and plant responses. In the presence of auxin, the degradation of Aux/IAA proteins is promoted after auxin is perceived by the receptor complex SCFTIR1/AFB in the nucleus, leading to the derepression of auxin response factors (ARFs) and the initiation of the auxin response [72,73,74,75]. The F box proteins TIR1 (Transport Inhibitor Response 1), AFB1, AFB2, and AFB3 assemble into SCF complexes and function as auxin receptors [74]. ARFs are transcription factors that have the capability of recognizing AUXIN RESPONSE ELEMENTs (AuxREs) within the promoter regions of downstream genes and regulating their expression [73,76]. Once the expression of *ARFs* appears to be de-repressed or activated, a subset of primary auxin response genes, such as *GH3 (Gretchen Hagen 3)* and *SAUR (Small auxin-up RNA)*, are activated or de-repressed [77]. As a result, this system functions to induce extremely rapid changes in response to auxin. In turn, the active auxin level in cells is regulated to match the needs of plant development.

Extensive studies have shown that the core components of the auxin signaling pathway function in the seed development of plants, especially during the development of embryos. The spatial pattern of auxin responses appears to be easily visualized by monitoring the synthetic auxin-responsive promoter DR5, which also allows detection of auxin redistribution [62]. The use of DR5rev::GFP reveals the dynamic gradients of auxin accumulation and response during early embryogenesis in Arabidopsis. Immediately after the division of the zygotes, the accumulation and response of auxin become detectable in the apical cell and then increase in the developing proembryo, with a weak signal in the suspensor [62]. In Arabidopsis, all auxin receptor genes are expressed during embryogenesis, among which the *TIR1* and *AFB1* genes are moderately expressed, whereas *AFB2* and *AFB3* are expressed at relatively high levels [74]. Compared with the normal embryos of *tir1-1*, *afb2-1*, and *afb3-1* single mutants, 48% of *tir1-1 afb2-1 afb3-1* embryos appear to have defects in embryogenesis [74]. Previous studies have also shown that Aux/IAA proteins might be involved in seed development. The phenotype of the Arabidopsis gain-of-function mutant *BODENLOSS (BDL)*, whose mutated gene encodes IAA12, is similar to that of the *mp* mutant and strongly resembles the phenotype of *tir1 afb* triple and quadruple mutant seedlings [78]. In addition, a previous study showed that *AtIAA18* is expressed in the apical domain of globular embryos [79]. Gain-of-function *iaa18-1* mutations maintain the stability of the Aux/IAA protein IAA18, leading to the formation of aberrant cotyledon placement in embryos of Arabidopsis [79]. To date, the roles of ARF transcription factors in embryo development have been well documented. Mutants of *ARF2*, *ARF3*, and *ARF5/MONOPTEROS (MP)* appear to have defects in Arabidopsis embryonic development, in which the *monopteros* mutant fails to initiate root meristem during early embryogenesis [15,78,80,81,82]. In Arabidopsis, *ARF6* and *ARF8* have been confirmed to be directly targeted by *microRNA167* (*miR167*) in regulating plant reproduction [83,84]. It has been demonstrated that *MIR167A*, acting as a maternal gene, modulates embryonic and endosperm development mainly by targeting *ARF6* and *ARF8* [85]. In addition, despite a lack of knowledge about the functional confirmation of *ZmARF* genes, seven *ZmARF* genes appear to exhibit constitutive expression patterns in developing embryos [86].

Moreover, auxin is also involved in other processes of seed development. By utilizing a *DR5v2::VENUS* reporter, researchers have shown that auxin signaling is active in seed coat development [42]. ARF2 regulates seed size by inhibiting integument cell proliferation during ovule development [15,82]. Furthermore, ARF3/ETTIN (ETT) physically interacts with KANADI (KAN) transcription factors to regulate integument development in Arabidopsis, which is required for embryo, leaf, carpel, and ovule development [87]. The alterations associated with the phenotype of *ett* mutants appear to be similar to those of the mutants of *ABERRANT TESTA SHAPE* (*ATS* or *KAN4*), which form abnormal seeds bearing congenital fusion of the inner and outer integuments [87]. In tobacco, the expression of *ARF* genes is modulated by NtTTG2 (TRANSPARENT TESTA GLABRA 2), which is required for the development of seeds [88]. Additional studies have shown that the expression of *NtTTG2* can induced by a synthetic auxin, 1-naphthaleneacetic acid (NAA), and that the function of *NtTTG2* in seed production is also associated with NtARF8 [89]. In addition, ARF18 regulates silique development by accelerating cell expansion in the silique walls of *Brassica napus* L., leading to changes in seed development [10].

## 5. Auxin Homeostasis-Mediated Regulation of Seed Development

It is well known that almost all aspects of the plant life cycle, including seed development, appear to be modulated by the concentration gradients of auxin, which are determined by the maintenance of the optimal active auxin level in different tissues and organs at different developmental stages [90,91,92]. The dynamic regulation of auxin homeostasis not only depends on auxin biosynthesis and polar transport but also modulates auxin conjugation (mainly to amino acids and sugars) and catabolism (mainly oxidation) [93,94,95]. Together with the above-mentioned data, there is a significant understanding of the contributions of auxin biosynthesis, polar transport, and signaling during seed development. However, although auxin conjugation and catabolism are well-known processes that take part in the dynamic regulation of auxin homeostasis, their roles in seed development remain less known due to a lack of identification of genes that are involved in auxin conjugation or catabolism and that modulate seed development. It is evident that the lack of suitable tools for quantifying and visualizing auxin metabolites at the cellular or tissue level hinders functional analyses of the roles of auxin conjugation and catabolism in seed development. Although many genes involved in auxin biosynthesis, transport, and signaling pathways have also been identified to be involved in the regulation of seed development, few genes modulate seed development by maintaining auxin homeostasis. However, indirect evidence suggests that auxin homeostasis is probably involved in seed development. For example, some signaling components involved in auxin signaling or conjugation, including genes encoding AUX/IAA proteins, ARFs, auxin-responsive SAUR family proteins and GH3 proteins, appear to be upregulated by MADS29, which is required for endosperm development in rice [54]. In line with this fact, in the defective *endosperm 18 (de18)* mutant of maize, the expression of key factors acting on the pathways controlling auxin homeostasis, such as *GH3* genes, a *DAO-like* (*DIOXYGENASE FOR AUXIN OXIDATION-like*) gene and 2 indole-3-acetate beta-glucosyltransferase genes, is significantly downregulated [50]. In addition, based on the analysis of differentially expressed genes the NIL (SW) (ARF18^-^) and R1 lines (ARF18^+^) of *Brassica napus*, it is of interest to see that additional early auxin-responsive genes, such as *Aux/IAA, SAUR,* and *GH3* genes, are differentially expressed [10].

At present, only *TGW6 (THOUSAND-GRAIN WEIGHT 6)*, which encodes a protein that exerts indole-3-acetic acid (IAA)-glucose hydrolase activity and controls IAA supplies, has been confirmed to be a negative regulator of grain weight and to increase rice yields [96]. Additional studies have shown that TGW6 affects the expression of many auxin-responsive genes, suggesting that TGW6 might be involved in regulating seed development in rice by modulating auxin homeostasis [96]. However, direct evidence to confirm the role of TGW6 at the cellular level of seed development and the mechanism by which TGW6 regulates auxin homeostasis are still lacking. 

**Figure 2:**
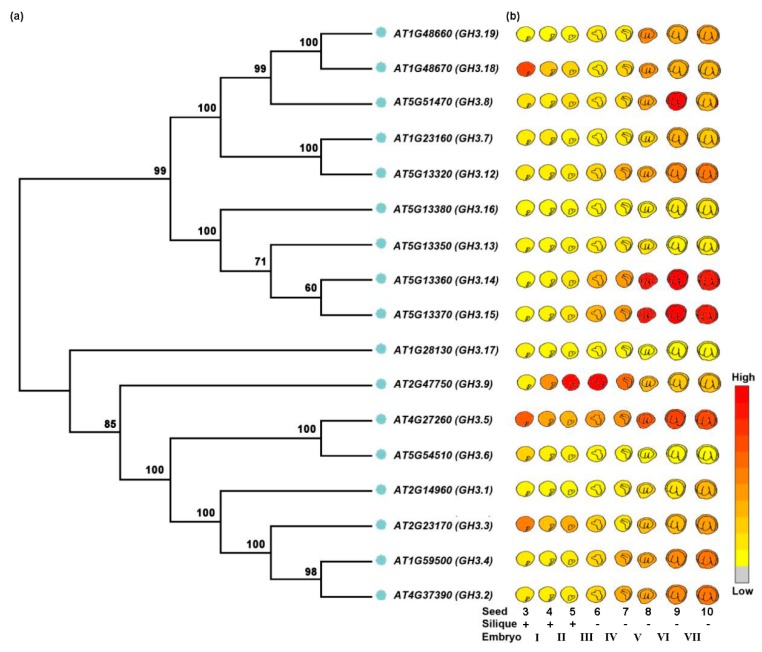
*GH3* sub-family genes involved in seed development in *Arabidopsis thaliana*. (**a**) Phylogenetic tree of the 17 Arabidopsis GH3 group II proteins. The tree was constructed using MEGA7.0 program by Neighbor Joining (NJ) clustering method. Bootstrap numbers (1000 replicates) are presented for all branches. (**b**) The expression pattern of Arabidopsis *GH3* group II genes during seed development. Data was collected from Arabidopsis eFP browser (http://bar.utoronto.ca/efp/cgi-bin/efpWeb.cgi?primaryGene). The experimental design can be downloaded from TAIR (The Arabidopsis Information Resource). Legend: I, globular; II, heart; III, torpedo; IV, walking-stick; V, VI, curled cotyledons; VII, green cotyledons.

Due to the lack of evidence concerning the role of auxin homeostasis during seed development, we attempted to analyse the expression patterns of *AtGH3* group II family gene members because of their crucial role in auxin homeostasis. Based on the data collected from the Arabidopsis eFP browser [97] and phylogenetic analyses of those proteins (Figure 2a,b; Supplementary Table S1; Supplemental data S2), it is apparent that these *AtGH3* homologous genes are differentially expressed during seed development. Most *AtGH3* genes, which could be divided into different clades (Figure 2a), appeared to be highly expressed during the late stages of seed development, except that *AtGH3.5* displays a more stable and abundant expression pattern throughout all stages of seed development (Figure 2b). Interestingly, some *GH3* genes having a closer phylogenetic relationship exhibit similar expression patterns during seed development (Figure 2a,b). For example, the *AtGH3.14* and *AtGH3.15* as well as the *AtGH3.2* and *AtGH3.4* gene pair shows higher levels of expression at the late stages of seed development than do other *AtGH3* members (Figure 2b). By contrast, other *GH3* genes with close evolutionary relationships, such as *AtGH3.18* and *AtGH3.19*, *AtGH3.7* and *AtGH3.12*, and *AtGH3.5* and *AtGH3.6*, show divergent expression patterns during seed development (Figure 2a,b). Furthermore, *AtGH3.6*, *AtGH3.13*, *AtGH3.16*, and *AtGH3.17*, which are divided into different evolutionary subclades (Figure 2a), are expressed at very low expression levels in seed development. Notably, although *AtGH3.3* and *AtGH3.18* are grouped into different clades (Figure 2a), both of them were highly expressed at both the early and late stages of seed development (Figure 2a,b). In contrast, *AtGH3.9* was specifically expressed at high levels in the middle stages of seed development. These results indicate that *GH3* family gene-mediated auxin homeostasis might regulate seed development. More effort is needed to develop imaging approaches and decipher the molecular regulatory mechanism of auxin homeostasis regulation of seed development.

## 6. Auxin Is a Main Factor Influencing Seed Size and Seed Weight of Crop Species

Seed development determines seed size and seed weight, which are the main traits of grain yields of crops. Due to the essential role of auxin in seed development, it is conceivable that auxin also functions as a key player in seed size and weight. Indeed, recent studies have shown that some auxin signaling components or genes regulated by the auxin signaling pathway can alter seed size and/or seed weight. For instance, ARF6 and ARF8, which are partially controlled by microR167, appear to be required for seed size in Camelina [98]. Big Grain 1 (BG1), which is a plasma membrane-associated protein involved in auxin transport and the auxin response, has the potential to improve both grain size and weight in rice [99]. Another QTL, *qTGW3* (*QTL FOR THOUSAND-GRAIN WEIGHT ON CHROMOSOME 3*), encoding the GSK3/SHAGGY-Like Kinase OsGSK5/OsSK41, negatively controls grain size and weight in rice by interacting with and phosphorylating OsARF4 [100], suggesting an important role of qTGW3 in auxin-regulated seed production. In *Brassica napus L., ARF18* has been identified based on forward genetics and has been confirmed to function in determining final seed weight by regulating the classic auxin signaling pathway during silique development [10]. In addition, some regulators associated with the auxin-mediated determination of seed size have also been identified. For instance, *SMOS1* (*SMALL ORGAN SIZE 1*) encodes an auxin-regulated APETAL2-type transcription factor and is positively regulated by OsARF1 in rice [101]. The loss of function of *SMOS1* causes pleiotropic developmental phenotypes, including small seed size [101,102], suggesting a crucial role for SMOS1 in seed size regulation.

Seed development is a complex and dynamic process that is strictly regulated by multiple growth regulators. It has been shown that auxin regulates seed development synergistically or antagonistically with other plant hormones. For example, SMOS1 can interact with the GRAS transcription factor SMOS2/DLT (DWARF AND LOW-TILLERING), which positively regulates seed development via brassinosteroid (BR) signaling to modulate its transcription activity. This finding provides the first evidence that the interplay of the auxin and BR signaling pathways modulates seed size in crops. Despite limited evidence for auxin-mediated seed size and seed weight, its indispensable role as a master regulator of seed development makes auxin a potential target for improving seed size, seed weight and grain yield in crop species.

## 7. Concluding Remarks: Auxin as a High Potential Target for the Optimization of Crop Yields

Seed size and seed weight regulated by seed development are critical determinants of crop yields. An improved understanding of the molecular mechanisms and regulatory networks underlying seed development will facilitate the elucidation of the genetic basis of yield-related traits and breeding of high-yielding crop species. Auxin is essential for optimal plant development and reproduction, but its role in seed development has received relatively little attention. Greater research efforts are needed to systemically decipher the molecular mechanisms of auxin-mediated seed development, especially the regulation of auxin homeostasis maintenance during seed development. In both model plant and crop species, the identification of additional critical genes related to auxin metabolism, transport, signaling or homeostasis is a key step in understanding the role of auxin during seed development. Additionally, elucidating the crosstalk between auxin and other signaling pathways is important to better understand the regulatory network underlying auxin control of seed development.

**Figure 3:**
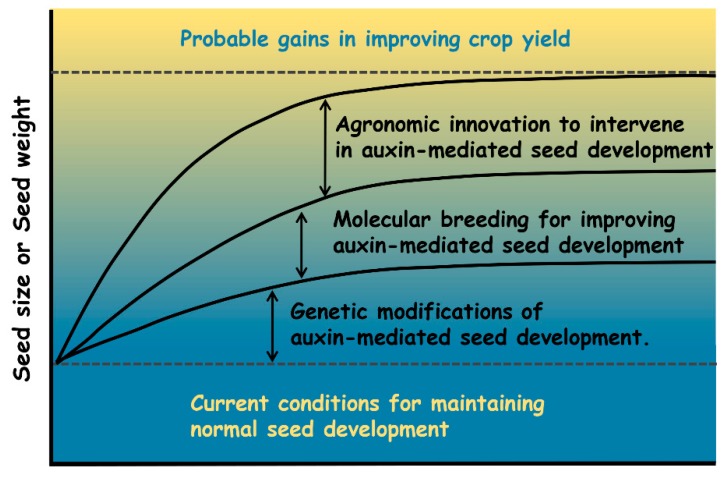
Potential strategies for future genetic improvement of crop yield.

Overall, this review has summarized the recent progress in auxin-mediated seed development, emphasizing the importance of auxin in regulating seed development and pinpointing the potential role of auxin in increasing crop yields. Fine tuning the auxin gradient and maxima may allow us to achieve desired crop performance and optimal yield. Based on the findings summarized in this review, we proposed three competitive strategies for increasing crop yields, listed here (Figure 3). (1) The first involves genetic modifications of auxin-mediated seed development. Once the auxin-related genes associated with seed traits are identified by reverse genetics or forward genetics, including QTL mapping or GWASs, combinatorial approaches for genetic modifications can be adopted. These include the overexpression and knock down (or the knockout) of the selected gene(s) and the specific modifications of gene expression driven by cell- or tissue-specific promoters using classic genetic methods or gene editing technology (e.g., the CRISPR/Cas9 system). (2) The second involves molecular breeding for improving auxin-mediated seed development. Molecular markers linked to the genes or QTLs controlling auxin-mediated seed development can be used to select improved plants with desired seed traits via marker-assisted breeding. (3) The third involves agronomic innovation to intervene in auxin-mediated seed development. Based on the role of auxin during seed development, exogenous applications of auxin or auxin transport inhibitors can be utilized to modulate the impact of auxin on seed development at specific stages of seed development in crops. In summary, combined with a deep understanding of the complex regulatory network underlying auxin-mediated seed development, advanced biotechnology for genetic manipulation, and appropriate agronomic innovation in a synergistic fashion, it might be anticipated that rewiring auxin homeostasis and cellular response governing seed development is a promising approach that allows us to increase crop seed production.

## Figures and Tables

**Table 1:** Identified genes involved in the auxin control of seed development in plants.

Botanical Classification	Species	Accession Number	Gene Name	Protein Category	Impact on Seed Development	Possible Role for Auxin	References
Monocotyledon	Maize	*GRMZM2G091819*	*ZmYuc1*	Flavin monooxygenases	Endosperm development	Involved in auxin biosynthesis	Bernardi et al., 2012
	Rice	*Os06g0623700*	*TGW6*	Indole-3-acetic acid IAA-glucose hydrolase	Cell number and grain length, Seed weight	Controls the supply of IAA	Ishimaru et al., 2013
	Rice	*Os02g07430*	*MADS29*	MADS-box transcription factor	Endosperm development	Induced by auxin	Yin and Xue, 2012
	Rice	*Os03g0175800*	*BG1*	Novel plasma membrane-associated protein	Grain size	Regulates auxin transport	Liu et al., 2015
	Rice	*Os05g32270*	*SMOS1*	AP2 transcription factor	Seed size	Induced by exogenous auxin treatment, interacts with ARF	Aya et al., 2014
	Rice	*Os07g0603700*	*OsGE/CYP78B5*	Cytochrome P450 enzyme	Embryo development	Regulates auxin responsive gene	Chen et al., 2014
	Rice	*Os03g62500*	*OsSK41*	GLYCOGEN SYNTHASE KINASE 3/SHAGGY-like family	Grain length, Grain weight	Interacts with OsARF4	Hu et al., 2018
	Rice	*Os06g03710*	*SMOS2/DLT*	GRAS transcription factor	Seed size	Involved in auxin–BR signaling crosstalk	Hirano et al., 2017
Dicotyledon	Pea	*JN990989*	*PsTAR2*	Trytophan aminotransferase related protein	Reduced starch content, Seed size	Involved in auxin biosynthesis	Tivendale et al., 2012; McAdam et al., 2017
	Rape	*BnaA09G55530D*	*BnaA9.CYP78A9*	Cytochrome P450 enzyme	Silique length, Seed size	Influences auxin metabolism or auxin biosynthesis	Shi et al., 2019
	Arabidopsis	*At4G32540, At5G11320, At1G48910, At1G21430*	*YUC1, YUC4, YUC10, YUC11*	Flavin monooxygenases	Embryogenesis and post-embryonic organ formation	Involved in auxin biosynthesis	Cheng et al., 2007
	Arabidopsis	*At1G28300*	*LEC2*	AP2/B3-like transcriptional factor family protein	Embryo development	Regulates the supply of auxin	Stone et al., 2008; Wójcikowska et al., 2013
	Arabidopsis	*At1G51950*	*IAA18*	Auxin-responsive protein	Cotyledon placement, Embryo growth	Interferes with auxin transport	Ploense et al., 2009
	Arabidopsis	*At2G38120, At5G01240, At2G21050*	*AUX1, LAX1, LAX2*	Transmembrane amino acid transporter family proteins	Endosperm development, Radicle apex growth	Regulates auxin transport	Robert et al., 2015; Ugartechea-Chirino et al., 2010
	Arabidopsis	*At5G60440*	*AGL62*	MADS-box transcription factor	Embryo development	Involved in auxin transport	Figueiredo et al., 2015
	Arabidopsis	*At1G73590, At1G70940, At2G01420, At1G23080*	*PIN1, PIN3, PIN4, PIN7*	PIN-FORMED proteins	Embryo development	Regulates auxin transport	Friml et al., 2003
	Medicago truncatula	*Medtr2g014060*	*DASH*	DOF transcription factor	Endosperm development	Affects auxin export	Noguero et al., 2015
	Arabidopsis	*At5G16560*	*KANADI*	Homeodomain-like superfamily protein	Integument development	Regulated by auxin	Kelley et al., 2012
	Arabidopsis	*At3G62980*	*TIR1*	F-box protein	Embryo development	Response to auxin	Dharmasiri et al., 2005
	Arabidopsis	*At4G03190, At3G26810, At1G12820*	*AFB1, AFB2, AFB3*	F-box proteins	Embryo development	Response to auxin	Dharmasiri et al., 2005
	Arabidopsis	*At5G62000*	*ARF2*	AUXIN RESPONSE FACTOR (ARF) transcription factor	Integument development, Seed size	Response to auxin	Schruff et al., 2006
	Arabidopsis	*At2G33860*	*ETT/ARF3*	AUXIN RESPONSE FACTOR (ARF) transcription factor	Integument development	Response to auxin	Kelley et al., 2012
	Arabidopsis	*At1G04550*	*BDL/IAA12*	AUXIN/INDOLE-3-ACETIC ACID (AUX/IAA) transcriptional repressors	Embryo development	Response to auxin	Hamann et al., 2002
	Arabidopsis	*At1G19850*	*MONOPTEROS/ARF5*	AUXIN RESPONSE FACTOR (ARF) transcription factor	Embryo development	Response to auxin	Berleth and Jürgens, 1993
	Arabidopsis	*At3G22886*	*MIR167A*	microRNA	Ovule development, Embryos growth, Endosperms development	Response to auxin	Yao et al., 2019; Na et al., 2019
	Rape	*BnaA09G55580D*	*ARF18*	AUXIN RESPONSE FACTOR (ARF) transcription factor	Silique development	Response to auxin	Liu et al., 2015
	Tobacco	*LOC107800718*	*NtTTG2*	WRKY transcription factor	Seed production, Seed development	Impacts the nuclear import of NtARF8	Zhu et al., 2013; Ge et al., 2016

## Supplementary Materials

Supplementary Materials can be found at https://www.mdpi.com/1422-0067/21/5/1662/s1.
